# Efficient algorithms for analyzing segmental duplications with deletions and inversions in genomes

**DOI:** 10.1186/1748-7188-5-11

**Published:** 2010-01-04

**Authors:** Crystal L Kahn, Shay Mozes, Benjamin J Raphael

**Affiliations:** 1 Department of Computer Science, Brown University, Providence, RI 02912, USA; 2 Center for Computational Molecular Biology, Brown University, Providence, RI 02912, USA

## Abstract

**Background:**

Segmental duplications, or low-copy repeats, are common in mammalian genomes. In the human genome, most segmental duplications are mosaics comprised of multiple duplicated fragments. This complex genomic organization complicates analysis of the evolutionary history of these sequences. One model proposed to explain this mosaic patterns is a model of repeated aggregation and subsequent duplication of genomic sequences.

**Results:**

We describe a polynomial-time exact algorithm to compute duplication distance, a genomic distance defined as the most parsimonious way to build a target string by repeatedly copying substrings of a fixed source string. This distance models the process of repeated aggregation and duplication. We also describe extensions of this distance to include certain types of substring deletions and inversions. Finally, we provide a description of a sequence of duplication events as a context-free grammar (CFG).

**Conclusion:**

These new genomic distances will permit more biologically realistic analyses of segmental duplications in genomes.

## Introduction

Genomes evolve via many types of mutations ranging in scale from single nucleotide mutations to large genome rearrangements. Computational models of these mutational processes allow researchers to derive similarity measures between genome sequences and to reconstruct evolutionary relationships between genomes. For example, considering chromosomal inversions as the only type of mutation leads to the so-called reversal distance problem of finding the minimum number of inversions/reversals that transform one genome into another [1]. Several elegant polynomial-time algorithms have been found to solve this problem (cf. [2] and references therein). Developing genome rearrangement models that are both biologically realistic *and *computationally tractable remains an active area of research.

Duplicated sequences in genomes present a particular challenge for genome rearrangement analysis and often make the underlying computational problems more difficult. For instance, computing reversal distance in genomes with duplicated segments is NP-hard [3]. Models that include both duplications and other types of mutations - such as inversions - often result in similarity measures that cannot be computed efficiently. Thus, most current approaches for duplication analysis rely on heuristics, approximation algorithms, or restricted models of duplication [3-7]. For example, there are efficient algorithms for computing tandem duplication histories [8-11] and whole-genome duplication histories [12,13]. Here we consider another class of duplications: large segmental duplications (also known as low-copy repeats) that are common in many mammalian genomes [14]. These segmental duplications can be quite large (up to hundreds of kilobases), but their evolutionary history remains poorly understood, particularly in primates. The mystery surrounding them is due in part to their complex organization; many segmental duplications are found within contiguous regions of the genome called *duplication blocks *that contain mosaic patterns of smaller repeated segments, or *duplicons *[15]. Duplication blocks that are located on different chromosomes, or that are separated by large physical distances on a chromosome, often share sequences of duplicons [16]. These conserved sequences suggest that these duplicons were copied together across large genomic distances. One hypothesis proposed to explain these conserved mosaic patterns is a two-step model of duplication [14]. In this model, a first phase of duplications copies duplicons from the ancestral genome and aggregates these copies into primary duplication blocks. Then in a second phase, portions of these primary duplication blocks are copied and reinserted into the genome at disparate loci forming secondary duplication blocks.

In [17], we introduced a measure called *duplication distance *that models the duplication of contiguous substrings over large genomic distances. We used duplication distance in [18] to find the most parsimonious duplication scenario consistent with the two-step model of segmental duplication. The duplication distance from a source string **x **to a target string **y **is the minimum number of substrings of **x **that can be sequentially copied from **x **and pasted into an initially empty string in order to construct **y**. We derived an efficient exact algorithm for computing the duplication distance between a pair of strings. Note that the string **x **does *not *change during the sequence of duplication events. Moreover, duplication distance does not model local rearrangements, like tandem duplications, deletions or inversions, that occur within a duplication block during its construction. While such local rearrangements undoubtedly occur in genome evolution, the duplication distance model focuses on identifying the duplicate operations that account for the construction of repeated patterns within duplication blocks by aggregating substrings of other duplication blocks over large genomic distances. Thus, like nearly every other genome rearrangement model, the duplication distance model makes some simplifying assumptions about the underlying biology to achieve computational tractability. Here, we extend the duplication distance measure to include certain types of deletions and inversions. These extensions make our model less restrictive - although we still maintain the restriction that **x **is unchanged - and permit the construction of more rich, and perhaps more biologically plausible, duplication scenarios. In particular, our contributions are the following.

### Summary of Contributions

Let *μ*(**x**) denote the number of times a character appears in the string **x**. Let |**x**| denote the length of **x**.

1. We provide an *O*(|**y**|^2^|**x**|*μ*(**x**) *μ*(**y**))-time algorithm to compute the distance between (signed) strings **x **and **y **when duplication and certain types of deletion operations are permitted.

2. We provide an *O*(|**y**|^2^*μ*(**x**) *μ*(**y**))-time algorithm to compute the distance between (signed) strings **x **and **y **when duplicated strings may be inverted before being inserted into the target string.

3. We provide an *O*(|**y**|^2^|**x**|*μ*(**x**)*μ*(**y**))-time algorithm to compute the distance between signed strings **x **and **y **when duplicated strings may be inverted before being inserted into the target string, and deletion operations are also permitted.

4. We provide an *O*(|**y**|^2^|**x**|^3^*μ*(**x**)*μ*(**y**))-time algorithm to compute the distance between signed strings **x **and **y **when any substring of the duplicated string may be inverted before being inserted into the target string. Deletion operations are also permitted.

5. We provide a formal proof of correctness of the duplication distance recurrence presented in [18]. No proof of correctness was previously given.

6. We show how a sequence of duplicate operations that generates a string can be described by a context-free grammar (CFG).

## Preliminaries

We begin by reviewing some definitions and notation that were introduced in [17] and [18]. Let ∅ denote the empty string. For a string **x **= *x*_1 _. . . *x*_*n*_, let **x**_*i*, *j *_denote the substring *x*_*i*_*x*_*i*+1 _. . . *x*_*j *_. We define a *subsequence S *of **x **to be a string  with *i*_1 _<*i*_2 _< ⋯ <*i*_*k*_. We represent *S *by listing the indices at which the characters of *S *occur in **x**. For example, if **x **= *abcdef*, then the subsequence *S *= (1, 3, 5) is the string *ace*. Note that every substring is a subsequence, but a subsequence need not be a substring since the characters comprising a subsequence need not be contiguous. For a pair of subsequences *S*_1_, *S*_2_, denote by *S*_1 _∩ *S*_2 _the maximal subsequence common to both *S*_1 _and *S*_2_.

**Definition 1**. *Subsequences S *= (*s*_1_, *s*_2_) *and T *= (*t*_1_, *t*_2_) *of a string **x **are **alternating **in **x **if either s*_1 _<*t*_1 _<*s*_2 _<*t*_2 _*or t*_1 _<*s*_1 _<*t*_2 _<*s*_2_.

**Figure 1:**
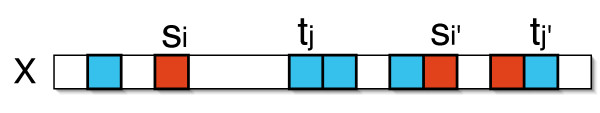
**Overlapping**. The red subsequence is overlapping with the blue subsequence in **x**. The indices (*s*_*i*_, *s*_*i*'_) and (*t*_*j*_, *t*_*j*'_) are alternating in **x**.

**Definition 2**. *Subsequences S *= (*s*_1_, . . ., *s*_*k*_) *and T *= (*t*_1_, . . ., *t*_*l*_) *of a string **x **are **overlapping **in **x **if there exist indices i, i' and j, j' such that *1 ≤ *i *<*i' *≤ *k*, 1 ≤ *j *<*j' *≤ l, *and *(*s*_*i*_, *s*_*i*'_) *and *(*t*_*j*_, *t*_*j*'_) *are alternating in **x**. See Figure *1.

**Figure 2:**
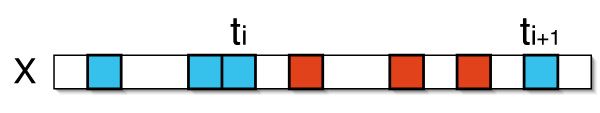
**Inside**. The red subsequence is inside the blue subsequence *T *. All the characters of the red subsequence occur between the indices *t*_*i *_and *t*_*i*+1 _of *T*.

**Definition 3**. *Given subsequences S *= (*s*_1_, . . ., *s*_*k*_) *and T *= (*t*_1_, . . ., *t*_*l*_) *of a string **x**, S is inside of T if there exists an index i such that *1 ≤ *i *<*l and t*_*i *_<*s*_1 _<*s*_*k *_<*t*_*i*+1_. *That is, the entire subsequence S occurs in between successive characters of T. See Figure *2.

**Figure 3:**
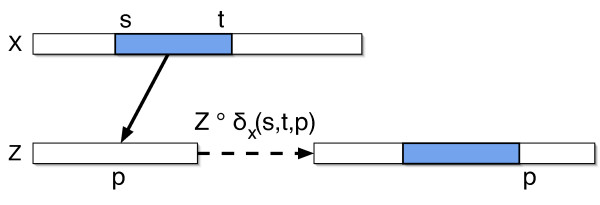
**A duplicate operation**. A duplicate operation, denoted *δ*_*x*_(*s, t, p*). A substring *x*_*s*_*x*_*s*+1 _. . *x*_*t *_of the source string **x **is copied and inserted into the target string **z **at index *p*.

**Definition 4**. *A **duplicate operation **from **x**, δ_*x*_*(*s, t, p*), *copies a substring x_*s *_*. . . *x*_*t *_*of the source string **x **and pastes it into a target string at position p. Specifically, if **x ***= *x*_1 _. . . *x*_*m *_*and **z ***= *z*_1 _. . . *z*_*n*_, *then **z ***∘ *δ*_*x*_(*s, t, p*) = *z*_1 _. . . *z*_*p*-1_*x*_*s *_. . . *x*_*t*_*z*_*p*_. . . *z*_*n*_. *See Figure *3.

**Definition 5**. *The **duplication distance **from a source string **x **to a target string **y **is the minimum number of duplicate operations from **x **that generates **y **from an initially empty target string. That is, **y ***= ∅ ∘ *δ*_*x*_(*s*_1_, *t*_1_, *p*_1_) ∘ *δ*_*x*_(*s*_2_, *t*_2_, *p*_2_) ∘ ⋯ ∘ *δ*_*x*_(*s*_*l*_, *t*_*l*_, *p*_*l*_).

To compute the duplication distance from **x **to **y**, we assume that every character in **y **appears at least once in **x**. Otherwise, the duplication distance is undefined.

## Duplication Distance

In this section we review the basic recurrence for computing duplication distance that was introduced in [18]. The recurrence examines the characters of the target string, **y**, and considers the sets of characters of **y **that could have been *generated*, or copied from the source string in a single duplicate operation. Such a set of characters of **y **necessarily correspond to a substring of the source **x **(see Def. 4). Moreover, these characters must be a subsequence of **y**. This is because, in a sequence of duplicate operations, once a string is copied and inserted into the target string, subsequent duplicate operations do not affect the order of the characters in the previously inserted string. Because every character of **y **is generated by exactly one duplicate operation, a sequence of duplicate operations that generates **y **partitions the characters of **y **into disjoint subsequences, each of which is generated in a single duplicate operation. A more interesting observation is that these subsequences are mutually non-overlapping. We formalize this property as follows.

**Lemma 1 (Non-overlapping Property)**. *Consider a source string **x **and a sequence of duplicate operations of the form δ*_*x*_(*s*_*i*_, *t*_*i*_, *p*_*i*_) *that generates the final target string **y **from an initially empty target string. The substrings **of **x **that are duplicated during the construction of **y **appear as mutually non-overlapping subsequences of **y***.

*Proof*. Consider a sequence of duplicate operations *δ*_*x*_(*s*_1_, *t*_1_, *p*_1_), . . ., *δ*_*x*_(*s*_*k*_, *t*_*k*_, *p*_*k*_) that generates **y **from an initially empty target string. For 1 ≤ *i *≤ *k*, Let **z**^*i *^be the intermediate target string that results from *δ*_*x*_(*s*_1_, *t*_1_, *p*_1_) ∘ ⋯ ∘ *δ*_*x*_(*s*_*i*_, *t*_*i*_, *p*_*i*_). Note that **z**^*k *^= **y**. For *j *≤ *i*, let  be the subsequence of **z**^*i *^that corresponds to the characters duplicated by the *j*^*th *^operation. We shall show by induction on the length *i *of the sequence that  are pairwise non-overlapping subsequences of **z**^*i*^. For the base case, when there is a single duplicate operation, there is no non-overlap property to show. Assume now that , . . .  are mutually non-overlapping subsequences in **z**^*i *-1^. For the induction step note that, by the definition of a duplicate operation,  is inserted as a contiguous substring into **z**^*i*-1 ^at location *p*_*i *_to form **z**^*i*^. Therefore, for any *j*, *j' *<*i*, if  and  are non overlapping in **z**^*i*-1 ^then  and , are non overlapping in **z**^*i*^. It remains to show that for any *j *<*i*,  and  are non-overlapping in **z**^*i*^. There are two cases: (1) the elements of  are either all smaller or all greater than the elements of  or (2)  is inside of  in **z**^*i *^(Definition 3). In either case,  and  are not overlapping in **z**^*i *^as required.

The non-overlapping property leads to an efficient recurrence that computes duplication distance. When considering subsequences of the final target string **y **that might have been generated in a single duplicate operation, we rely on the non-overlapping property to identify substrings of **y **that can be treated as independent subproblems. If we assume that some subsequence *S *of **y **is produced in a single duplicate operation, then we know that all other subsequences of **y **that correspond to duplicate operations cannot overlap the characters in *S*. Therefore, the substrings of **y **in between successive characters of *S *define subproblems that are computed independently.

In order to find the optimal (i.e. minimum) sequence of duplicate operations that generate **y**, we must consider all subsequences of **y **that could have been generated by a single duplicate operation. The recurrence is based on the observation that *y*_1 _must be the first (i.e. leftmost) character to be copied from **x **in some duplicate operation. There are then two cases to consider: either (1) *y*_1 _was the last (or rightmost) character in the substring that was duplicated from **x **to generate *y*_1_, or (2) *y*_1 _was not the last character in the substring that was duplicated from **x **to generate *y*_1_.

The recurrence defines two quantities: *d*(**x**, **y**) and *d*_*i*_(**x**, **y**). We shall show, by induction, that for a pair of strings, **x **and **y**, the value *d*(**x**, **y**) is equal to the duplication distance from **x **to **y **and that *d*_*i*_(**x**, **y**) is equal to the duplication distance from **x **to **y **under the restriction that the character *y*_1 _is copied from index *i *in **x**, i.e. *x*_*i *_*generates y*_1_. *d*(**x**, **y**) is found by considering the minimum among all characters *x*_*i *_of **x **that can generate *y*_1_, see Eq. 1.

**Figure 4:**
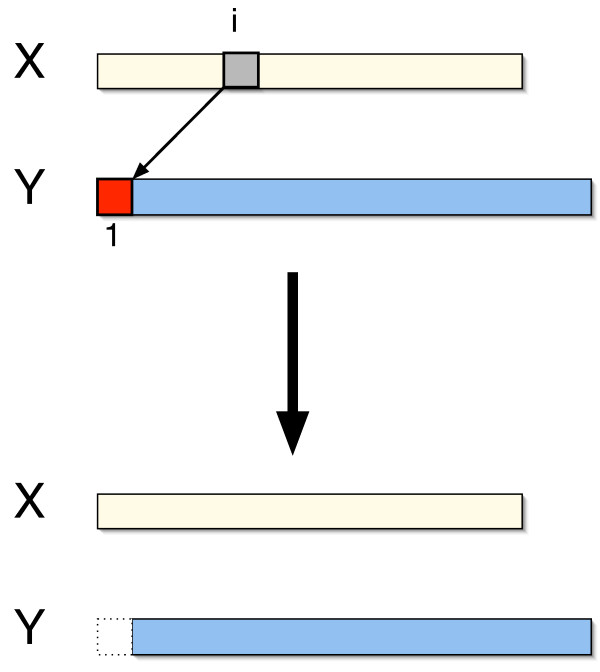
**Recurrence: Case 1**. *y*_1 _is generated from *x*_*i *_in a duplicate operation where *y*_1 _is the last (rightmost) character in the copied substring (Case 1). The total duplication distance is one plus the duplication distance for the suffix **y**_2,|**y**|_.

As described above, we must consider two possibilities in order to compute *d*_*i*_(**x**, **y**). Either:

Case 1: *y*_1 _was the last (or rightmost) character in the substring of **x **that was copied to produce *y*_1_, (see Fig. 4), or

**Figure 5:**
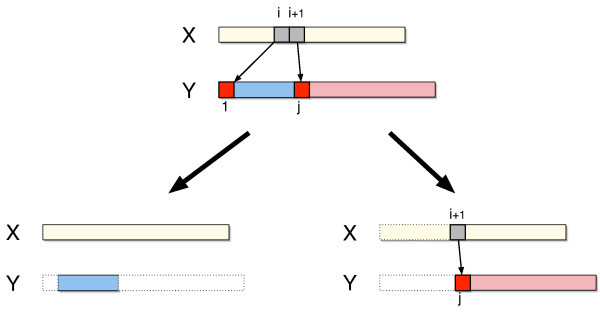
**Recurrence: Case 2**. *y*_1 _is generated from *x*_*i *_in a duplicate operation where *y*_1 _is not the last (rightmost) character in a copied substring (Case 2). In this case, *x*_*i*+1 _is also copied in the same duplicate operation (top). Thus, the duplication distance is the sum of *d*(**x**, **y**_2, *j*-1_), the duplication distance for **y**_2, *j*-1 _(bottom left), and *d*_*i*+1_(**x**, **y**_*j*, |**y**|_), the minimum number of duplicate operations to generate **y**_*j*, |**y**| _given that *x*_*i*+1 _generates *y*_*j *_(bottom right).

Case 2: *x*_*i*+1 _is also copied in the same duplicate operation as *x*_*i*_, possibly along with other characters as well (see Fig. 5).

For case one, the minimum number of duplicate operations is one - for the duplicate that generates *y*_1 _- plus the minimum number of duplicate operations to generate the suffix of **y**, giving a total of 1 + *d*(**x**, **y**_2,|**y**|_) (Fig. 4). For case two, Lemma 1 implies that the minimum number of duplicate operations is the sum of the optimal numbers of operations for two independent subproblems. Specifically, for each *j *> 1 such that *x*_*i*+1 _= *y*_*j *_we compute: (i) the minimum number of duplicate operations needed to build the substring **y**_2, *j*-1_, namely *d*(**x**, **y**_2, *j*-1_), and (ii) the minimum number of duplicate operations needed to build the string *y*_1_**y**_*j*,|**y**|_, given that *y*_1 _is generated by *x*_*i *_and *y*_*j *_is generated by *x*_*i*+1_. To compute the latter, recall that since *x*_*i*_and *x*_*i*+1 _are copied in the same duplicate operation, the number of duplicates necessary to generate *y*_1_**y**_*j*,|**y**| _using *x*_*i *_and *x*_*i*+1 _is equal to the number of duplicates necessary to generate **y**_*j*,|**y**| _using *x*_*i*+1_, namely *d*_*i*+1_(**x**, **y**_*j*,|**y**|_), (see Fig. 5 and Eq. 2).

The recurrence is, therefore:(1)

**Theorem 1**. *d*(***x, y***) *is the minimum number of duplicate operations that generate **y **from **x***. *For *{*i *: *x*_*i *_= *y*_1_}, *d*_*i*_(***x***, ***y***) *is the minimum number of duplicate operations that generate **y **from **x **such that y*_1 _*is generated by x*_*i*_.

*Proof*. Let *OPT*(**x**, **y**) denote minimum length of a sequence of duplicate operations that generate **y **from **x**. Let *OPT*_*i*_(**x**, **y**) denote the minimum length of a sequence of operations that generate **y **from **x **such that *y*_1 _is generated by *x*_*i*_. We prove by induction on |**y**| that *d*(**x**, **y**) = *OPT*(**x**, **y**) and *d*_*i*_(**x**, **y**) = *OPT*_*i*_(**x**, **y**).

For |**y**| = 1, since we assume there is at least one *i *for which *x*_*i *_= *y*_1_, *OPT *(**x**, **y**) = *OPT*_*i*_(**x**, **y**) = 1. By definition, the recurrence also evaluates to 1. For the inductive step, assume that *OPT *(**x**, **y**') = *d*(**x**, **y**') and *OPT*_*i*_(**x**, **y**') = *d*_*i*_(**x**, **y'**) for any string **y' **shorter than **y**. We first show that *OPT*_*i*_(**x**, **y**) ≤ *d*_*i*_(**x**, **y**). Since *OPT *(**x**, **y**) = min_*i *_*OPT*_*i*_(**x**, **y**), this also implies *OPT *(**x**, **y**) ≤ *d*(**x**, **y**). We describe different sequences of duplicate operations that generate **y **from **x**, using *x*_*i *_to generate *y*_1_:

• Consider a minimum-length sequence of duplicates that generates **y**_2,|**y**|_. By the inductive hypothesis its length is *d*(**x**, **y**_2,|**y**|_). By duplicating *y*_1 _separately using *x*_*i *_we obtain a sequence of duplicates that generates **y **whose length is 1 + *d*(**x**, **y**_2,|**y**|_).

• For every {*j *: *y*_*j *_= *x*_*i*+1_, *j *> 1} consider a minimum-length sequence of duplicates that generates **y**_*j*,|**y**| _using *x*_*i*+1 _to produce *y*_*j*_, and a minimum-length sequence of duplicates that generates **y**_2, *j*-1_.

By the inductive hypothesis their lengths are *d*_*i*+1_(**x**, **y**_*j*,|**y**|_) and *d*(**x**, **y**_2, *j*-1_) respectively. By extending the start index *s *of the duplicate operation that starts with *x*_*i*+1 _to produce *y*_*j *_to start with *x*_*i *_and produce *y*_1 _as well, we produce **y **with the same number of duplicate operations.

Since *OPT*_*i*_(**x**, **y**) is at most the length of any of these options, it is also at most their minimum. Hence,

To show the other direction (i.e. that *d*(*x, y*) ≤ *OPT *(*x, y*) and *d*_*i*_(*x, y*) ≤ *OPT*_*i*_(*x, y*)), consider a minimum-length sequence of duplicate operations that generate **y **from **x**, using *x*_*i *_to generate *y*_1_. There are a few cases:

• If *y*_1 _is generated by a duplicate operation that only duplicates *x*_*i*_, then *OPT*_*i*_(**x**, **y**) = 1 + *OPT *(**x**, **y**_2,|**y**|_). By the inductive hypothesis this equals 1 + *d*(**x**, **y**_2,|**y**|_) which is at least *d*_*i*_(**x**, **y**).

• Otherwise, *y*_1 _is generated by a duplicate operation that copies *x*_*i *_and also duplicates *x*_*i*+1 _to generate some character *y*_*j *_. In this case the sequence Δ of duplicates that generates **y**_2, *j*-1 _must appear after the duplicate operation that generates *y*_1 _and *y*_*j *_because **y**_2, *j*-1 _is inside (Definition 3) of (*y*_1_, *y*_*j*_). Without loss of generality, suppose Δ is ordered after all the other duplicates so that first *y*_1_*y*_*j *_. . . *y*_|**y**| _is generated, and then Δ generates *y*_2 _. . . *y*_*j*-1 _between *y*_1 _and *y*_*j *_. Hence, *OPT*_*i*_(**x**, **y**) = *OPT*_*i*_(**x**, *y*_1_**y**_*j*,|**y**|_) + *OPT *(**x**, *y*_2, *j*-1_). Since in the optimal sequence *x*_*i *_generates *y*_1 _in the same duplicate operation that generates *y*_*j *_from *x*_*i*+1_, we have *OPT*_*i*_(**x**, *y*_1_**y**_*j*,|**y**|_) = *OPT*_*i*+1_(**x**, **y**_*j*,|**y**|_). By the inductive hypothesis, *OPT *(**x**, **y**_2, *j*-1_) + *OPT*_*i*+1_(**x**, **y**_*j*,|**y**|_) = *d*(**x**, **y**_2, *j*-1_) + *d*_*i*+1_(**x**, **y**_*j*,|**y**|_) which is at least *d*_*i*_(**x**, **y**).   □

This recurrence naturally translates into a dynamic programing algorithm that computes the values of *d*(**x**, *·*) and *d*_*i*_(**x**, *·*) for various target strings. To analyze the running time of this algorithm, note that both **y**_2, *j *_and **y**_*j*,|**y**| _are substrings of **y**. Since the set of substrings of **y **is closed under taking substrings, we only encounter substrings of **y**. Also note that since *i *is chosen from the set {*i *: *x*_*i *_= *y*_1_}, there are *O*(*μ*(**x**)) choices for *i*, where *μ*(**x**) is the maximal multiplicity of a character in **x**. Thus, there are *O*(*μ*(**x**)|**y**|^2^) different values to compute. Each value is computed by considering the minimization over at most *μ*(**y**) previously computed values, so the total running time is bounded by *O*(|**y**|^2^*μ*(**x**)*μ*(**y**)), which is *O*(|**y**|^3^|*x*|) in the worst case. As with most dynamic programming approaches, this algorithm (and all others presented in subsequent sections) can be extended through trace-back to reconstruct the optimal sequence of operations needed to build **y**. We omit the details.

Extending to Affine Duplication Cost

It is easy to extend the recurrence relations in Eqs. (1), (2) to handle costs for duplicate operations. In the above discussion, the cost of each duplicate operation is 1, so the sum of costs of the operations in a sequence that generates a string **y **is just the length of that sequence. We next consider a more general cost model for duplication in which the cost of a duplicate operation *δ*_*x*_(*s, t, p*) is Δ_1 _+ (*t - s *+ 1) Δ_2 _(i.e., the cost is affine in the number of duplicated characters). Here Δ_1_, Δ_2 _are some non-negative constants. This extension is obtained by assigning a cost of Δ_2 _to each duplicated character, except for the last character in the duplicated string, which is assigned a cost of Δ_1 _+ Δ_2_. We do that by adding a cost term to each of the cases in Eq. 2. If *x*_*i*_is the last character in the duplicated string (case 1), we add Δ_1 _+ Δ_2 _to the cost. Otherwise *x*_*i *_is not the last duplicated character (case 2), so we add just Δ_2 _to the cost. Eq. (2) thus becomes(3)

The running time analysis for this recurrence is the same as for the one with unit duplication cost.

## Duplication-Deletion Distance

In this section we generalize the model to include deletions. Consider the intermediate string **z **generated after some number of duplicate operations. A deletion operation removes a contiguous substring *z*_*i*_, . . ., *z*_*j *_of **z**, and subsequent duplicate and deletion operations are applied to the resulting string.

**Figure 6:**
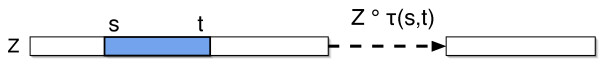
**A delete operation**. A delete operation, denoted *t *(*s, t*). The substring **z**_*s*, *t *_is deleted.

**Definition 6**. *A **delete operation**, τ *(*s, t*), *deletes a substring z*_*s *_. . . *z*_*t *_*of the target string **z**, thus making **z **shorter. Specifically, if **z ***= *z*_1 _. . . *z*_*s *_. . . *z*_*t *_. . . *z*_*m*_, *then **z ***∘ *τ *(*s, t*) = *z*_1 _. . . *z*_*s*-1_*z*_*t*+1 _. . . *z*_*m*_. *See Figure *6.

The cost associated with *t *(*s, t*) depends on the number *t - s *+ 1 of characters deleted and is denoted Φ(*t - s *+ 1).

**Definition 7**. *The **duplication-deletion **distance from a source string **x **to a target string **y **is the cost of a minimum sequence of duplicate operations from **x **and deletion operations, in any order, that generates **y***.

We now show that although we allow arbitrary deletions from the intermediate string, it suffices to consider deletions from the duplicated strings before they are pasted into the intermediate string, provided that the cost function for deletion, Φ(·) is non-decreasing and obeys the triangle inequality.

**Definition 8**. *A **duplicate-delete **operation from **x**, η*_*x*_(*i*_1_, *j*_1_, *i*_2_, *j*_2_,. . ., *i*_*k*_, *j*_*k*_, *p*), *for i*_1 _≤ *j*_1 _<*i*_2 _≤ *j*_2 _< ⋯ <*i*_*k *_≤ *j*_*k *_*copies the subsequence **of the source string **x **and pastes it into a target string at position p. Specifically, if **x ***= *x*_1 _. . . *x*_*m *_*and **z ***= *z*_1 _. . . *z*_*n*_, *then **z **∘ η*_*x*_(*i*_1_, *j*_1_, . . ., *i*_*k*_, *j*_*k*_, *p*) = .

The cost associated with such a duplication-deletion is Δ_1 _+ (*j*_*k *_- *i*_1 _+ 1)Δ_2 _+ . The first two terms in the cost reflect the affine cost of duplicating an entire substring of length *j*_*k *_- *i*_1 _+ 1, and the second term reflects the cost of deletions made to that substrings.

**Lemma 2**. *If the affine cost for duplications is non-decreasing and *Φ (·) *is non-decreasing and obeys the triangle inequality then the cost of a minimum sequence of duplicate and delete operations that generates a target string **y **from a source string **x **is equal to the cost of a minimum sequence of duplicate-delete operations that generates **y **from **x***.

*Proof*. Since duplicate operations are a special case of duplicate-delete operations, the cost of a minimal sequence of duplicate-delete operations and delete operations that generates **y **cannot be more than that of a sequence of just duplicate operations and delete operations. We show the (stronger) claim that an arbitrary sequence of duplicate-delete and delete operations that produces a string **y **with cost *c *can be transformed into a sequence of just duplicate-delete operations that generates **y **with cost at most *c *by induction on the number of delete operations. The base case, where the number of deletions is zero, is trivial. Consider the first delete operation, *τ *. Let *k *denote the number of duplicate-delete operations that precede *τ*, and let **z **be the intermediate string produced by these *k *operations. For *i *= 1, . . ., *k*, let *S*_*i *_be the subsequence of **x **that was used in the *i*th duplicate-delete operation. By lemma 1, *S*_1_, . . ., *S*_*k *_form a partition of **z **into disjoint, non-overlapping subsequences of **z**. Let *d *denote the substring of **z **to be deleted. Since *d *is a contiguous substring, *S*_*i *_∩ *d *is a (possibly empty) substring of *S*_*i *_for each *i*. There are several cases:

1. *S*_*i *_∩ *d *= ∅. In this case we do not change any operation.

2. *S*_*i *_∩ *d *= *S*_*i*_. In this case all characters produced by the *i*th duplicate-delete operation are deleted, so we may omit the *i*th operation altogether and decrease the number of characters deleted by *τ *. Since Φ (·) is non-decreasing, this does not increase the cost of generating **z **(and hence **y**).

3. *S*_*i *_∩ *d *is a prefix (or suffix) of *S*_*i*_. Assume it is a prefix. The case of suffix is similar. Instead of deleting the characters *S*_*i *_∩ *d *we can avoid generating them in the first place. Let *r *be the smallest index in *S*_*i*_\*d *(that is, the first character in *S*_*i *_that is not deleted by *τ*). We change the *i*th duplicate-delete operation to start at *r *and decrease the number of characters deleted by *τ *. Since the affine cost for duplications is non-decreasing and Φ (·) is non-decreasing, the cost of generating **z **does not increase.

4. *S*_*i *_∩ *d *is a non-empty substring of *S*_*i *_that is neither a prefix nor a suffix of *S*_*i*_. We claim that this case applies to at most one value of *i*. This implies that after taking care of all the other cases *τ *only deletes characters in *S*_*i*_. We then change the *i*th duplicate-delete operation to also delete the characters deleted by *τ*, and omit *τ *. Since Φ (·) obeys the triangle inequality, this will not increase the total cost of deletion. By the inductive hypothesis, the rest of **y **can be generated by just duplicate-delete operations with at most the same cost. It remains to prove the claim. Recall that the set {*S*_*i*_} is comprised of mutually non-overlapping subsequences of **z**. Suppose that there exist indices *i *≠ *j *such that *S*_*i *_∩ *d *is a non-prefix/suffix substring of *S*_*i *_and *S*_*j *_∩ *d *is a non-prefix/suffix substring of *S*_*j *_. There must exist indices of both *S*_*i *_and *S*_*j *_in **z **that precede *d*, are contained in *d*, and succeed *d*. Let *i*_*p *_<*i*_*c *_<*i*_*s *_be three such indices of *S*_*i *_and let *j*_*p *_<*j*_*c *_<*j*_*s *_be similar for *S*_*j *_. It must be the case also that *j*_*p *_<*i*_*c *_<*j*_*s *_and *i*_*p *_<*j*_*c *_<*i*_*s*_. Without loss of generality, suppose *i*_*p *_<*j*_*p*_. It follows that (*i*_*p*_, *i*_*c*_) and (*j*_*p*_, *j*_*s*_) are alternating in **z**. So, *S*_*i *_and *S*_*j*_are overlapping which contradicts Lemma 1.

To extend the recurrence from the previous section to duplication-deletion distance, we must observe that because we allow deletions in the string that is duplicated from **x**, if we assume character *x*_*i *_is copied to produce *y*_1_, it may not be the case that the character *x*_*i*+1 _also appears in **y**; the character *x*_*i*+1 _may have been deleted. Therefore, we minimize over all possible locations *k *>*i *for the next character in the duplicated string that is not deleted. The extension of the recurrence from the previous section to duplication-deletion distance is:(4)

**Theorem 2**. (***x***, ***y***) *is the duplication-deletion distance from **x **to **y**. For *{*i *: *x*_*i *_= *y*_1_}, (***x***, ***y***) *is the duplication-deletion distance from **x **to **y **under the additional restriction that y*_1_*is generated by x*_*i*_.

The proof of Theorem 2 is almost identical to that of Theorem 1 in the previous section and is omitted. However, the running time increases; while the number of entries in the dynamic programming table does not change, the time to compute each entry is multiplied by the possible values of *k *in the recurrence, which is *O*(|**x**|). Therefore, the running time is *O*(|**y**|^2^|**x**|*μ*(**x**)*μ*(**y**)), which is *O*(|**y**|^3^|**x**|^2^) in the worst case. We conclude this section by showing, in the following lemma, that if both the duplicate and delete cost functions are the identity function (i.e. one per operation), then the duplication-deletion distance is equal to duplication distance without deletions.

**Lemma 3**. *Given a source string **x**, a target string **y**, If the cost of duplication is 1 per duplicate operation, and the cost of deletion is 1 per delete operation, then *(***x***, ***y***) = *d*(***x***, ***y***).

*Proof*. First we note that if a target string **y **can be built from **x **in *d*(**x**, **y**) duplicate operations, then the same sequence of duplicate operations is a valid sequence of duplicate and delete operations as well, so *d*(**x**, **y**) is at least (**x**, **y**).

We claim that every sequence of duplicate and delete operations can be transformed into a sequence of duplicate operations of the same length. The proof of this claim is similar to that of Lemma 2. In that proof we showed how to transform a sequence of duplicate and delete operations into a sequence of duplicate-delete operations of at most the same cost. We follow the same steps, but transform the sequence into an a sequence that consists of just duplicate operations without increasing the number of operations. Recall the four cases in the proof of Lemma 2. In the the first three cases we eliminate the delete operation without increasing the number of duplicate operations. Therefore we only need to consider the last case (*S*_*i *_∩ *d *is a non-empty substring of *S*_*i *_that is neither a prefix nor a suffix of *S*_*i*_). Recall that this case applies to at most one value of *i*. Deleting *S*_*i *_∩ *d *from *S*_*i *_leaves a prefix and a suffix of *S*_*i*_. We can therefore replace the *i*^*th *^duplicate operation and the delete operation with two duplicate operations, one generating the appropriate prefix of *S*_*i *_and the other generating the appropriate suffix of *S*_*i*_. This eliminates the delete operation without changing the number of operations in the sequence. Therefore, for any string **y **that results from a sequence of duplicate and delete operations, we can construct the same string using only duplicate operations (without deletes) using at most the same number of operations. So, *d*(**x**, **y**) is no greater than (**x**, **y**).

## Duplication-Inversion Distance

In this section we extend the duplication-deletion distance recurrence to allow inversions. We now explicitly define characters and strings as having two orientations: forward (+) and inverse (-).

**Definition 9**. *A **signed string **of length m over an alphabet *Σ *is an element of *({+, *-*} *× *Σ)^*m*^.

For example, (+*b -c -a *+*d*) is a signed string of length 4. An inversion of a signed string reverses the order of the characters as well as their signs. Formally,

**Definition 10**. *The **inverse **of a signed string **x ***= *x*_1 _. . . *x*_*m *_*is a signed string * = *-x*_*m *_. . . -*x*_1_.

For example, the inverse of (+*b -c -a *+*d*) is (*-d *+*a *+*c -b*).

In a duplicate-invert operation a substring is copied from **x **and *inverted *before being inserted into the target string **y**. We allow the cost of inversion to be an affine function in the length ℓ of the duplicated inverted string, which we denote Θ_1 _+ ℓΘ_2_, where Θ_1_, Θ_2 _≥ 0. We still allow for normal duplicate operations.

**Definition 11**. *A **duplicate-invert operation **from **x***, (*s, t, p*), *copies an inverted substring -x*_*t*_, -*x*_*t*_*-*_1 _. . ., -*x*_*s *_*of the source string **x **and pastes it into a target string at position p. Specifically, if **x ***= *x*_1 _. . . *x*_*m *_*and **z ***= *z*_1 _. . . *z*_*n*_, *then **z ***∘ (*s, t, p*) = .

The cost associated with each duplicate-invert operation is Θ_1_+ (*t *- *s *+ 1)Θ_2_.

**Definition 12**. *The **duplication-inversion distance **from a source string **x **to a target string **y **is the cost of a minimum sequence of duplicate and duplicate-invert operations from **x**, in any order, that generates **y***.

The recurrence for duplication distance (Eqs. 1, 3) can be extended to compute the duplication-inversion distance. This is done by introducing a term for inverted duplications whose form is very similar to that of the term for regular duplication (Eq. 3). Specifically, when considering the possible characters to generate *y*_1_, we consider characters in **x **that match either *y*_1 _or its inverse, -*y*_1_. In the former case, then, we use (**x**, **y**) to denote the duplication-inversion distance with the additional restriction that *y*_1 _is generated by *x*_*i *_without an inversion. The recurrence for  is the same as for *d*_*i *_in Eq. 3. In the latter case, we consider an inverted duplicate in which *y*_1 _is generated by -*x*_*i*_. This is denoted by , which follows a similar recurrence. In this recurrence, since an inversion occurs, *x*_*i *_is the *last *character of the duplicated string, rather than the first one. Therefore, the next character in **x **to be used in this operation is *-x*_*i*-1 _rather than *x*_*i*+1_. The recurrence for  also differs in the cost term, where we use the affine cost of the duplicate-invert operation. The extension of the recurrence to duplication-inversion distance is therefore:(6)

**Theorem 3**. (***x***, ***y***) *is the duplication-inversion distance from **x **to **y**. For *{*i *: *x*_*i *_= *y*_1_},  (***x***, ***y***) *is the duplication-inversion distance from **x **to **y **under the additional restriction that y*_1 _*is generated by x*_*i*_. *For *{*i *: *x*_*i *_= *-y*_1_},  (***x***, ***y***) *is the duplication-inversion distance from **x **to **y **under the additional restriction that y*_1_*is generated by -x*_*i*_.

The correctness proof is very similar to that of Theorem 1, only requiring an additional case for handling the case of a duplicate invert operation which is symmetric to the case of regular duplication. The asymptotic running time of the corresponding dynamic programming algorithm is *O*(|**y**|^2^*μ*(**x**)*μ*(**y**)). The analysis is identical to the one in section 3. The fact that we now consider either a duplicate or a duplicate-invert operation does not change the asymptotic running time.

## Duplication-Inversion-Deletion Distance

In this section we extend the distance measure to include delete operations as well as duplicate and duplicate-invert operations. Note that we only handle deletions after inversions of the same substring. The order of operations might be important, at least in terms of costs. The cost of inverting (+*a *+*b *+*c*) and then deleting *-b *may be different than the cost of first deleting +*b *from (+*a *+*b *+*c*) and then inverting (+*a *+*c*).

**Definition 13**. *The **duplication-inversion-deletion distance **from a source string **x **to a target string **y **is the cost of a minimum sequence of duplicate and duplicate-invert operations from **x **and deletion operations, in any order, that generates **y***.

**Definition 14**. *A **duplicate-invert-delete **operation from **x***,

(*i*_1_, *j*_1_, *i*_2_, *j*_2_, . . ., *i*_*k*_, *j*_*k*_, *p*), *for i*_1 _≤ *j*_1 _<*i*_2 _≤ *j*_2 _*<*⋯ <*i*_*k*_≤ *j*_*k *_*pastes the string **into a target string at position p. Specifically, if **x ***= *x*_1 _. . . *x*_*m *_*and **z ***= *z*_1 _. . . *z*_*n*_, *then **z ***∘ (*i*_1_, *j*_1_, *i*_2_, *j*_2_, . . ., *i*_*k*_, *j*_*k*_, *p*) = .

The cost of such an operation is Θ_1 _+ (*j*_*k *_- *i*_1 _+ 1)Θ_2 _+ . Similar to the previous section, it suffices to consider just duplicate-invert-delete and duplicate-delete operations, rather than duplicate, duplicate-invert and delete operations.

**Lemma 4**. *If *Φ (·) *is non-decreasing and obeys the triangle inequality and if the cost of inversion is an affine non-decreasing function as defined above, then the cost of a minimum sequence of duplicate, duplicate-invert and delete operations that generates a target string **y **from a source string **x **is equal to the cost of a minimum sequence of duplicate-delete and duplicate-invert-delete operations that generates **y **from **x***.

The proof of the lemma is essentially the same as that of Lemma 2. Note that in that proof we did not require all duplicate operations to be from the same string **x**. Therefore, the arguments in that proof apply to our case, where we can regard some of the duplicates from **x **and some from the inverse of **x**.

The recurrence for duplication-inversion-deletion distance is obtained by combining the recurrences for duplication-deletion (Eq. 5) and for duplication-inversion distance (Eq. 6). We use separate terms for duplicate-delete operations () and for duplicate-invert-delete operations (). Those terms differ from the terms in Eq. 6 in the same way Eq. 5 differs from Eq. 2; Because of the possible deletion we do not know that *x*_*i*+1 _(*x*_*i*-1_) is the next duplicated character. Instead we minimize over all characters later (earlier) than *x*_*i*_.

The recurrence for duplication-inversion-deletion distance is therefore:

**Theorem 4**. (***x***, ***y***) *is the duplication-inversion-deletion distance from **x **to **y***. *For *{*i *:*x*_*i *_= *y*_1_},  (***x***, ***y***) *is the duplication-inversion-deletion distance from **x **to **y **under the additional restriction that y*_1 _*is generated by x*_*i*_. *For *{*i *: *x*_*i *_= *-y*_1_},  (***x***, ***y***) *is the duplication-inversion-deletion distance from **x **to **y **under the additional restriction that y*_1_*is generated by -x*_*i*_.

The proof, again, is very similar to the proofs in the previous sections. The running time of the corresponding dynamic programming algorithm is the same (asymptotically) as that of duplication-deletion distance. It is *O*(|**y**|^2^|**x**|*μ*(**y**)*μ*(**x**)), where the multiplicity *μ*(**y**) (or *μ*(**x**)) is the number of times a character appears in the string **y **(or **x**), regardless of its sign.

In comparing the models of the previous section and the current one, we note that restricting the model of rearrangement to allow only duplicate and duplicate-invert operations (Section 5) instead of duplicate-invert-delete operations may be desirable from a biological perspective because each duplicate and duplicate-invert requires only three breakpoints in the genome, whereas a duplicate-invert-delete operation can be significantly more complicated, requiring more breakpoints.

## Variants of Duplication-Inversion-Deletion Distance

It is possible to extend the model even further. We give here one detailed example which demonstrates how such extensions might be achieved. Other extensions are also possible. In the previous section we handled the model where the duplicated substring of **x **may be inverted in its entirety before being inserted into the target string. In the generalized model a substring of the duplicated string may be inverted before the string is inserted into **y**. For example, we allow (+*a *+*b *+*c *+*d *+*e *+*f*) to become (+*a *+*b -e -d -c *+*f*) before being inserted into **y**. In this model, the cost of duplicating a string of length *m *with an inversion of a substring of length ℓ is Δ_1 _+ *m*Δ_2 _+ Θ (ℓ), for some non-negative monotonically increasing cost function Θ.

The way we extend the recurrence is by considering all possible substring inversions to the original string **x**. For 1 ≤ *s ≤ t ≤ |x|*, let  be the string *x*_1 _. . . *x*_*s*-1 _-*x*_*t*_. . . -*x*_*s *_*x*_*t*+1 _. . . *x*_|**x**|_. That is, the string that is obtained from **x **by inverting (in-place) **x**_*s*, *t*_. For convenience, define also  = **x**. We will use  (**x**, **y**) to denote the distance from **x **to **y **in this model under the additional restriction that *y*_1 _is generated by *x*_*i *_and that the substring **x**_*s*, *t *_was inverted. Note that this does not make much sense unless *s *≤ *i *≤ *t*, since otherwise the inverted substring is not used in the duplication. However, restricting the inversion cost Θ (ℓ) to be non-negative and monotonically increasing makes sure that those cases will not contribute to the minimization since inverting a character that is not duplicated will only increase the cost. The recurrence for duplication-deletion with arbitrary-substring-duplicate-inversions distance is given below.

The running time is *O*(|**y**|^2^|**x**|^3^*μ*(**x**)*μ*(**y**)). The multiplicative |**x**|^2 ^factor in the running time in comparison with that of the previous section arises from considering all possible inverted substrings of **x**. We note that if we were only interested in handling inversions to just a prefix or a suffix of the duplicated string, then it is possible to extend the duplication-inversion-deletion recurrence without increasing the asymptotic running time.

## Duplication Distance as a Context-Free Grammar

The process of generating a string **y **by repeatedly copying substings of a source string **x **and pasting them into an initially empty target string is naturally described by a context-free grammar (CFG). This alternative view might be useful in understanding our algorithms and their correctness. Thus, we provide the basic idea behind this connection for the most simple variant of duplication distance: no inversions or deletions and the cost of each duplicate operation is 1. For a fixed source string **x**, we construct a grammar *G*_*x *_in which for every *i, j *such that 1 ≤ *i *≤ *j *≤ |**x**|, there is a production rule *S → Sx*_*i*_*Sx*_*i*+1_*S . . . Sx*_*j*_*S*.

These production rules correspond to duplicating the substring **x**_*i*, *j *_. In addition there is a trivial production rule *S *→ ∈, where ∈ denotes the empty string. It is easy to see that the language described by this grammar is exactly the set of strings that can be duplicated from **x**. The non-overlapping property (Lemma 1) is now an immediate consequence of the structure of parse trees of CFGs. Finding the duplication distance from **x **to **y **is equivalent to finding a parse tree with a minimal number of non-trivial productions among all possible parse trees for **y**.

Consider now the slightly different grammar obtained by removing the leading *S *to the left of *x*_*i *_from each of the production rules, so that the new rules are of the form *S → x*_*i*_*Sx*_*i*+1_*S . . . Sx*_*j *_*S*. It is not difficult to see that both grammars produce the same language and have the same minimal size parse tree for every string **y**. The change only restricts the order in which rules are applied. For example, *y*_1 _is always produced by the first production rule.

**Figure 7:**
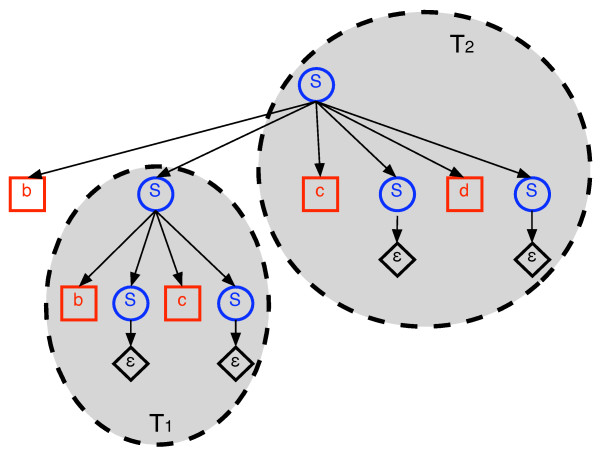
**Example parse tree**. An optimal parse tree *T *for **y **= bbccd where **x **= abcd. The root production duplicates **x**_2,4 _= bcd. *x*_2 _generates *y*_1 _and *x*_3 _generates *y*_4_. The trees *T*_1 _and *T*_2 _are indicated. *T*_1 _is an optimal parse tree for **y**_2,4-1 _= bc. *T*_2 _is an optimal parse tree for **y**_4,|**y**| _= cd.

**Figure 8:**
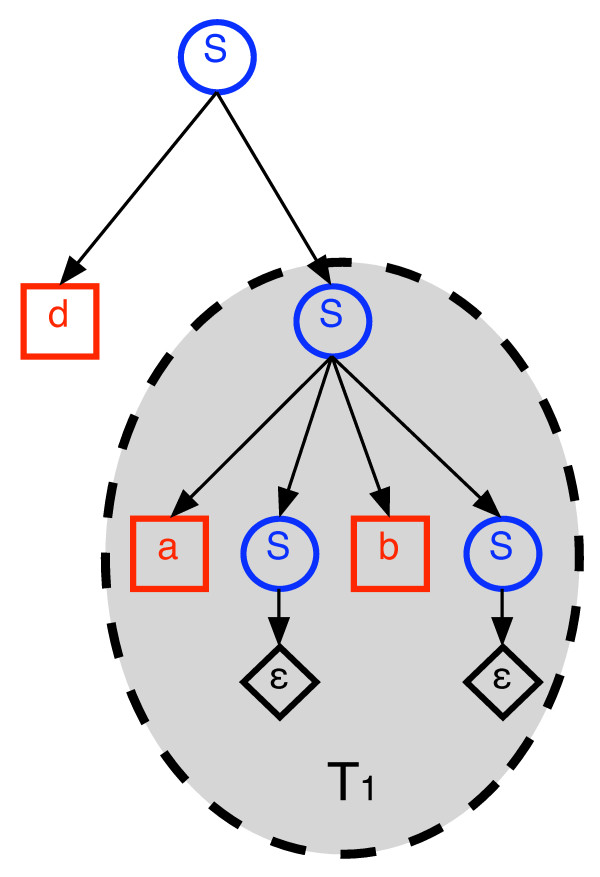
**Example parse tree**. An optimal parse tree *T *for **y **= dab where **x **= abcd. The root production duplicates just *x*_4 _= d. The tree *T*_1 _is indicated. *T*_2 _is empty (not indicated). The root production is not part of *T*_1_.

The recurrence for *d*_*i*_(**x**, **y**) naturally arises by observing that if *T *is an optimal parse tree for **y **in which the first production rule generates *y*_1 _by *x*_*i *_and *y*_*j *_by *x*_*i*+1_, then the subtree *T*_1 _of *T *that generates **y**_2, *j*-1 _is a valid parse tree which is optimal for **y **_2, *j*-1_. Similarly, the tree *T*_2 _obtained by deleting *x*_*i *_and *T*_1 _from *T *is a valid parse tree which is optimal for **y**_*j*,|**y**| _under the restriction that *y*_*j *_must be generated by *x*_*i*+1 _(see Fig. 7). Moreover, *T*_1 _and *T*_2 _are disjoint trees which contain all non trivial productions in *T *. This explains the term *d*(**x**, **y**_2, *j*-1_) + *d*_*i*+1_(**x**, **y**_*j*,|**y**|_) in Eq. 2, which is the heart of the recursion. The minimization over {*j *: *y*_*j *_= *x*_*i*+1_, *j *> 1} simply enumerates all of the possibilities for constructing *T *. The term 1 + *d*(**x**, **y**_2,|**y**|_) handles the possibility that *y*_1 _is generated by a duplicate operation that ends with *x*_*i*_. In this case the tree *T*_2 _is empty, so we only consider *T*_1_. We add one to account for the production rule at the root of *T *which is not part of *T*_1_. This is illustrated in Fig. 8.

## Conclusion

We have shown how to generalize duplication distance to include certain types of deletions and inversions and how to compute these new distances efficiently via dynamic programming. In earlier work [17,18], we used duplication distance to derive phylogenetic relationships between human segmental duplications. We plan to apply the generalized distances introduced here to the same data to determine if these richer computational models yield new biological insights.

## Competing interests

The authors declare that they have no competing interests.

## Authors' contributions

CLK, SM, and BJR all designed and analyzed the algorithms and drafted the manuscript. All authors read and approved the final manuscript.
